# Learning Domain-Invariant Representations for Event-Based Motion Segmentation: An Unsupervised Domain Adaptation Approach

**DOI:** 10.3390/jimaging11110377

**Published:** 2025-10-27

**Authors:** Mohammed Jeryo, Ahad Harati

**Affiliations:** Department of Computer Engineering, Faculty of Engineering, Ferdowsi University of Mashhad (FUM), Mashhad 9177948974, Iran; mo.jeryo@mail.um.ac.ir

**Keywords:** motion segmentation, event camera, unsupervised domain adaptation, cross-modality learning, real-time inference

## Abstract

Event cameras provide microsecond temporal resolution, high dynamic range, and low latency by asynchronously capturing per-pixel luminance changes, thereby introducing a novel sensing paradigm. These advantages render them well-suited for high-speed applications such as autonomous vehicles and dynamic environments. Nevertheless, the sparsity of event data and the absence of dense annotations are significant obstacles to supervised learning for motion segmentation from event streams. Domain adaptation is also challenging due to the considerable domain shift in intensity images. To address these challenges, we propose a two-phase cross-modality adaptation framework that translates motion segmentation knowledge from labeled RGB-flow data to unlabeled event streams. A dual-branch encoder extracts modality-specific motion and appearance features from RGB and optical flow in the source domain. Using reconstruction networks, event voxel grids are converted into pseudo-image and pseudo-flow modalities in the target domain. These modalities are subsequently re-encoded using frozen RGB-trained encoders. Multi-level consistency losses are implemented on features, predictions, and outputs to enforce domain alignment. Our design enables the model to acquire domain-invariant, semantically rich features through the use of shallow architectures, thereby reducing training costs and facilitating real-time inference with a lightweight prediction path. The proposed architecture, alongside the utilized hybrid loss function, effectively bridges the domain and modality gap. We evaluate our method on two challenging benchmarks: EVIMO2, which incorporates real-world dynamics, high-speed motion, illumination variation, and multiple independently moving objects; and MOD++, which features complex object dynamics, collisions, and dense 1kHz supervision in synthetic scenes. The proposed UDA framework achieves 83.1% and 79.4% accuracy on EVIMO2 and MOD++, respectively, outperforming existing state-of-the-art approaches, such as EV-Transfer and SHOT, by up to 3.6%. Additionally, it is lighter and faster and also delivers enhanced mIoU and F1 Score.

## 1. Introduction

Motion segmentation is a core computer vision task that separates a sequence of image views and partitions them into areas having coherent motion based on similarities in their motion patterns. Note that the observer (e.g., a camera or robot) itself can be moving, adding another layer of complexity to segmentation. It finds its usage as an integral part of numerous applications such as video compression and coding [1], scene interpretation and annotation [2], augmented reality [3], human–computer interaction [4], avoidance of obstacles [5], and recent robotic systems like autonomous vehicles [6] and unmanned aerial vehicles [7].

Modern autonomous systems, including self-driving cars, drones, and aerial robots, operate in dynamic, unstructured, and unpredictable conditions that are difficult to forecast. These systems must quickly understand their surroundings and identify moving objects in real-time. This is crucial for safely navigating around people, objects, and other robots, especially in challenging conditions such as changing lighting, rapid motion, and obstacles. Because of this, standard monocular, frame-based approaches often don’t work as well in these situations [8,9,10,11] because they depend on strict assumptions about scene geometry, item classifications, or specific motion models [12,13,14]. In addition, frame-based sensors have built-in problems that make it much harder to accurately estimate motion, such as motion blur, low temporal resolution, and sensitivity to changes in lighting. These limitations make it clear that we need new sensory modalities that can accurately capture complex changes across time.

Event cameras, which are based on the principles of biological vision, have become powerful sensors for detecting motion in complex and dynamic environments. This has led to the creation and use of advanced neuromorphic devices like the iniVation DAVIS346 [15] in modern robotics and automotive systems. Event cameras work differently from traditional cameras because they don’t take pictures at fixed frame rates. Instead, they detect changes in brightness at the pixel level in a logarithmic way. An event is triggered whenever the change in intensity goes above a certain contrast level [16]. This creates a steady, sparse stream of events, each of which contains information about its location, polarity, and a timestamp accurate to the microsecond. This asynchronous design gives you data with very low latency and high temporal resolution, which lets you accurately capture fast motion without blur and with as little data redundancy as possible. Additionally, their high dynamic range (HDR) enables them to perform well in both very bright and very dark conditions, and their low power consumption makes them suitable for platforms with limited resources. These features make event cameras a very useful way to sense motion for tasks like dense motion segmentation [17], long-term trajectory estimation [18], and real-time motion perception [19] in robotics and autonomous systems that work in difficult environments.

Existing event-based motion segmentation methods, particularly optimization-based approaches [20,21,22,23,24,25,26,27,28,29,30,31] remain limited in their applicability to real-world scenarios. Though these methods don’t involve ground-truth labeling, these methods incur computationally costly optimization over entire event streams due to high latency, non-real-time inference, and limited generalization. Such methods thus erase any efficiency benefit provided by event cameras and restrict their usefulness in robotics and other dynamically challenging environments. Compared with optimization-based methods, deep learning methods provide an encouraging alternative by offering fast feed-forward inference through the expressive capacity of deep neural networks (DNNs). Thus, a number of work [5,17,32,33,34,35,36,37,38,39] used encoder–decoder-type architectures [40] to promote efficient segmentation of independently moving objects (IMOs) from sparse event streams. Nevertheless, their performance relies strongly on large-scale, densely annotated datasets. In the context of motion segmentation, obtaining accurate pixel-level annotations is especially challenging due to the asynchronous and sparse nature of event data, the need for dedicated hardware, and the misalignment issues caused by mismatched temporal resolutions between sensors. Furthermore, event-based datasets are limited, constituting merely 3.14% of the total available visual data [41], which presents a considerable obstacle to the development of strong models. The lack of high-quality annotations significantly constrains learning efficacy, especially in dense segmentation tasks. Besides annotation scarcity, event-based motion segmentation presents modality-specific problems. Because event cameras only signal per-pixel changes in illumination intensity, these generate a sparse edge-centric event-based representation without rich texture information. This sparsity makes it challenging for encoder–decoder architectures to predict dense motion masks, particularly in regions with low motion or resolution. Additionally, standard pixel-wise loss functions (e.g., cross-entropy) can’t tap fully the rich temporal dynamics inscribed in event streams. Another critical limitation is that of domain shift, so that supervised models overfit their training domain. For instance, models trained on RGB data with clean ground-truth masks generally do not transfer well to new domains with different motion dynamics, lighting conditions, or sensing modalities, such as event cameras. Thus, there is a critical need for learning paradigms that avoid or minimize reliance on labeled data and enable models to transfer more easily and efficiently to new domains.

Unsupervised Domain Adaptation (UDA) [42] has become a potent and extensively utilized learning framework for transferring information from a labeled source domain to an unlabeled target domain without necessitating manual supervision in the latter. In motion segmentation, an effective approach is to utilize annotated RGB frames and optical flow from the source domain to guide learning on unlabelled event streams in the target domain. The fundamental concept of UDA is to alleviate domain disparity by mapping data from both the source and target domains into a common, domain-invariant latent space, therefore aligning their distributions [43,44]. This common representation enables models trained on the labeled source domain to generalize proficiently to the unlabeled target domain. A key advantage of UDA is its ability to eliminate the need for costly pixel-level annotations in the target domain. Furthermore, it enhances model generalization to novel settings and sensing modalities, thereby improving adaptability in real-world deployment scenarios. Despite fundamental differences in sensing mechanisms, frame-based and event-based cameras capture the same scenes, allowing meaningful cross-domain correspondence. However, transferring models trained on frame-based RGB data to the event domain remains challenging due to modality-specific characteristics and substantial distributional discrepancies. UDA mitigates this challenge through domain discriminators in adversarial learning, or by directly aligning source and target features using metric minimization and enforcing consistency constraints across domains. Collectively, these techniques of alignment and domain-invariant representation learning augment model robustness, especially in situations when ground-truth annotations are scarce or nonexistent [45]. Furthermore, UDA promotes cross-modality learning by amalgamating complementary signals from diverse sensors, including appearance-rich RGB and motion-sensitive event data, into a cohesive picture. This capability makes UDA an essential component in modern cross-domain visual segmentation frameworks, particularly those designed for deployment in resource-constrained or real-time environments. Although event-based and frame-based cameras operate through fundamentally different sensing mechanisms, they capture complementary information about the same physical dynamics in the real world [44]. This underlying correlation has sparked growing interest in using Unsupervised Domain Adaptation (UDA) to bridge the modality gap in event-based vision tasks.

Initial event-based domain adaptation techniques depend on spatially and temporally synchronized RGB-event data [41,46], facilitating direct supervision across modalities. The necessity for synchronized acquisition confines their use to controlled settings and hinders generalization to real-world situations. Subsequent methodologies have adopted reconstruction-based strategies to surmount these synchronization restrictions. Prominent techniques like E2VID [47] and VID2E [48,49] utilise generative models to transform RGB frames into synthetic events, facilitating cross-modal learning through intermediate representations. Nonetheless, these models often demonstrate diminished performance when utilized on real-world event streams, chiefly because of substantial domain discrepancies between synthetic and actual data distributions. A subsequent UDA framework, EV-Transfer [50], attempted to bridge the modality gap by hallucinating motion from a static image to generate pseudo-events (fake-events), which were then used to align latent spaces of image and event modalities via adversarial learning. However, the inherent uncertainty of the motion synthesis process limited the effectiveness of alignment and undermined representation stability. The ESS framework [43] applies UDA by aligning motion-invariant event embeddings with image embeddings through event-to-image reconstruction. While effective for transferring semantic segmentation, it suffers from blurred reconstructions and noisy pseudo-labels due to the lack of texture in event data. Furthermore, the use of a hard alignment process may introduce artifacts that compromise segmentation performance. More recent methods [45] explore domain adversarial training, self-training with pseudo-labels, and contrastive representation learning, which aim to learn domain-invariant features without requiring paired data. These developments mark significant progress, but a fully effective and unified solution for dense prediction in the event domain, particularly one that can harness the strengths of both appearance and motion modalities, remains an open challenge. Conversely, our proposed method distinctly separates appearance and motion representations via dual-encoder architectures, facilitating more stable and interpretable alignment. Our method efficiently overcomes the cross-modality divide by translating asynchronous event streams into pseudo-RGB and pseudo-flow modalities, while utilizing multi-level consistency across spatial and temporal domains, all without the necessity of paired data or target labels. This design directly tackles the shortcomings of previous research concerning reconstruction noise, representational instability, and the entanglement of motion-content features, providing a cohesive and resilient framework for dense event-based motion segmentation.

**Figure 1:**
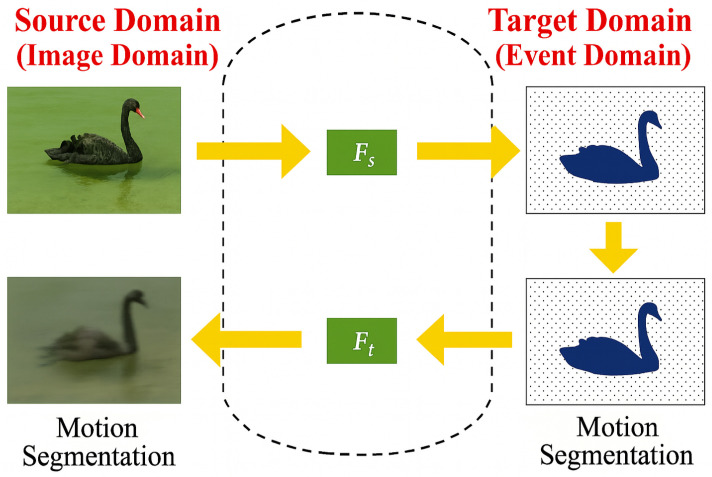
Conceptual overview of the proposed two-phase cross-modality adaptation pipeline. The model transfers motion segmentation knowledge from a labeled RGB-flow source domain to an unlabeled event-based target domain through pseudo-modality reconstruction followed by feature alignment via multi-level consistency losses.

Figure 1 provides a high-level overview of our proposed framework, which aims to perform motion segmentation in an unlabeled event-based target domain by transferring knowledge from a labeled RGB-flow source domain. The left side of the pipeline illustrates the supervised learning setup, in which RGB images and optical flow are processed through dedicated appearance and motion encoders. The extracted features are fused and passed to a segmentation head, guided by pixel-wise annotated masks in the source domain. In contrast, the right side of the figure depicts the target domain, where asynchronous event streams are encoded using a dedicated event encoder. From the latent event representation, pseudo-RGB and pseudo-flow reconstructions are produced and re-encoded utilizing the identical encoders applied in the source domain. A multi-level fusion and consistency approach synchronizes latent representations across domains, facilitating precise segmentation of event streams without requiring manual annotations. The framework effectively utilizes cross-modality reconstruction, temporal consistency, and domain adaptation techniques to bridge the modality and distribution gap between source and target domains, facilitating robust motion comprehension in real-world scenarios characterized by rapid motion and difficult lighting conditions.

To this end, we propose a novel two-phase cross-modality adaptation framework designed for unsupervised motion segmentation in the event domain. In the source domain, labeled RGB images and optical flow are processed by a dual-stream encoder that learns to disentangle and integrate motion and appearance cues. In the target domain, asynchronous event voxel grids are transformed into pseudo-RGB and pseudo-flow modalities using dedicated reconstruction networks. These pseudo-modalities are then re-encoded using frozen RGB-trained encoders, enabling consistent feature representations across domains. Domain alignment is enforced through multi-level consistency losses across features, predictions, and segmentation outputs. Importantly, our framework enables domain-invariant representation learning across both spatial (appearance) and temporal (motion) modalities. This allows accurate inference in the target domain without requiring any paired data or ground-truth labels.

We validate the proposed approach through extensive experiments on two challenging benchmarks: EVIMO2 contains real-world event streams with rapid object motion and dynamic lighting, and MOD++, a synthetic dataset featuring dense supervision and highly dynamic multi-object scenes. Our method consistently outperforms existing unsupervised domain adaptation (UDA) baselines, including reconstruction-based and adversarial alignment methods, in the context of event-based motion segmentation. Our methodology attains an accuracy of 83.1% on EVIMO2 and 79.4% on MOD++, reflecting absolute improvements of up to 3.6% above previous state-of-the-art techniques, including EV-Transfer and SHOT. These results illustrate the resilience of our system across both synthetic and real-world modalities, and affirm its significant generalisation capability under domain shifts and sparse input situations.

The main contributions of this work are summarized as follows:

- **Two-phase cross-modality adaptation:** We propose the first two-phase cross-modality domain adaptation framework for event-based independent motion segmentation, which transfers task knowledge from labeled RGB-flow data to unlabeled event streams. This design enables unsupervised learning without requiring paired data or target labels, even in complex motion scenarios.
- **Dual domain-invariant representation learning:** We offer a dual-pathway architecture that learns disentangled domain-invariant representations for appearance and motion in event streams. The proposed methodology mitigates representational interference by separating spatial and temporal inputs into distinct encoding streams, thereby enhancing latent space alignment and improving generalization across diverse sensing modalities.
- **Multi-level consistency for robust domain alignment:** We develop a hierarchical consistency framework that enforces alignment across various levels, specifically feature embeddings, task predictions, and segmentation outputs. This technique improves representational stability and facilitates precise motion segmentation by aligning both low-level and high-level representations to a common, domain-invariant structure without requiring target supervision.

The remainder of this paper is organized as follows. Section 2 reviews the related work on event-based motion segmentation and unsupervised domain adaptation methods applied to event-based vision tasks. Section 3 describes the preliminaries and problem formulation underlying our proposed method. In Section 4, we go into great detail about our proposed framework, including its architecture, loss functions, and training strategy. Section 5 talks about how the experiments were set up and what happened with both synthetic and real-world event-based benchmarks. We compare our method to the best supervised motion segmentation algorithms and unsupervised domain adaptation methods by giving both quantitative and qualitative assessments. Section 6 talks about an ablation study that looks at how much each module improves the proposed method. Finally, Section 7 sums up the paper and talks about where it might go in the future.

## 2. Related Work

### 2.1. Event-Based Motion Segmentation Methods

Event-based motion segmentation techniques can be broadly categorized into optimization-based and learning-based approaches, each offering distinct trade-offs in terms of accuracy, efficiency, and generalizability. Optimization-based techniques predict motion parameters and segmentation labels by optimizing task-dependent objectives, typically rooted in maximizing contrast or compensating for motion. Glover et al. [20,21] proposed a technique for identifying circular objects in chaotic environments utilizing a directed Hough transform informed by optical flow, subsequently refined with particle filtering to augment temporal stability. Nevertheless, these methodologies depend on robust geometric priors, which constrain their generalizability. However, they are based on strong geometry prior information, which restricts the level of generalizability. Vasco et al. [22] considered independent motion detection based on the calibration of prior information through robot joint information, followed by the clustering of corner motions, but the approach is based on assumptions of the motion-geometric relationship. More recent optimization-based approaches adopt motion compensation techniques [51] to handle motion segmentation in dynamic scenes captured by moving cameras. Stoffregen et al. [23] introduced a two-degree-of-freedom optical flow model (2-DoF) to progressively eliminate background motion. Mitrokhin et al. [24] proposed a 4-DoF model utilizing average timestamps and Kalman filtering for the segmentation and tracking of independently moving objects; however, it encountered difficulties in complicated scenarios due to its reliance on threshold-based grouping. Stoffregen et al. [25] redefined the segmentation task as an Expectation-Maximization (EM) problem, concurrently estimating labels and motion parameters by contrast maximization, while necessitating previous knowledge of the item count. Parameshwara et al. [26] introduced MOMS, a reliable approach that does not rely on object quantity or structure. Global motion correction, K-means clustering, and iterative merging and refinement are used on residual events. Their follow-up work [27] enhanced runtime with motion propagation and key-slice algorithms, and introduced the MOD++ dataset for complex motion patterns like collisions. Zhou et al. [28] introduced EMSGC, a graph-based method that uses motion-compensated graph cuts to segment events. Lu et al. [29] further this concept by implementing a cascaded two-stage system that integrates progressive motion fitting with 3D Markov Random Field (MRF) inference. Chen et al. [30] developed ProgressiveMotionSeg, combining motion estimation and event denoising in a mutually reinforced iterative loop. Their framework handles noisy event data by jointly refining motion clusters and suppressing background activity. More recently, Yamaki et al. [31] developed a variational contrast maximization method that updates motion parameters and segmentation labels without assuming IMO number, resulting in effective results in indoor and outdoor scenes. Optimization-based algorithms work directly on raw event streams and are independent of big labeled datasets. They have various drawbacks: Global optimization over entire event streams is computationally expensive, limiting real-time applicability; local minima and degenerate solutions are vulnerable; strong priors like motion models or object count are required; and hyperparameter tuning reduces generalization across domains. These restrictions have prompted interest in learning-based alternatives to improve scalability, adaptability, and deployment.

Recent learning-based approaches have achieved promising results in event-based motion segmentation by leveraging spatiotemporal representations learned directly from raw event streams. Mitrokhin et al. [32] made one of the initial efforts by proposing a compositional pipeline that concurrently predicts depth, 6-DoF camera motion, individual object 3D velocities, and per-pixel segmentation through a motion-compensated ECN-based network [52]. The EV-IMO dataset was also introduced, comprising ground-truth motion masks and depth for benchmarking purposes. Subsequently, GConvNet [33] was investigated for graph-based learning, which models long-term scene dynamics utilizing unstructured graphs to infer motion, facilitating occlusion-aware reasoning independent of LSTMs. Sanket et al. [5] introduced EVDodgeNet, a lightweight network trained semi-supervised on synthetic data, demonstrating real-time segmentation and obstacle avoidance in robotic navigation on resource-constrained platforms. Parameshwara et al. [34] presented SpikeMS, a supervised motion segmentation framework that utilizes spiking neural networks (SNNs) and incorporates novel spike-based loss functions and backpropagation techniques; however, training is complicated by the non-differentiability of spike activations. To address multi-object motion, FusionSeg [35] employed a dual encoder-decoder design that integrates frame and event modalities, utilizing attention mechanisms for object-specific differentiation. Nonetheless, it fails to adequately exploit the detailed temporal granularity of event data. MSRNN [36] tackles this issue by implementing a multi-scale recurrent architecture that collects both local and global motion signals across time, trained on the ECMotion dataset, which encompasses real-world complications like as occlusions and dense motions. Chenao Jiang et al. [37] developed a semantic-aided network to concurrently execute motion and semantic segmentation, facilitating improved multi-class object differentiation within a streamlined architecture. Concurrently, Yusra Alkendi et al. [17] introduced GTNN, a graph-transformer model that utilises self-attention on 3D event graphs, attaining precise segmentation without the necessity for explicit geometric priors or temporal alignment. Zhang et al. [38] introduced a modular event-based motion segmentation system to tackle generalization issues in intricate environments, separating flow reconstruction from dynamic segmentation. Zhang et al. [39] recently proposed an instance-level motion segmentation approach that combines image and event modalities via dual embeddings, capturing appearance through image textures and motion confidence through event dynamics. Their cross-modal design utilizes masked augmentation and contrastive learning across frames to extract distinctive characteristics, while a flow-guided refinement module further improves motion representations, facilitating robust segmentation in various indoor and outdoor contexts.

Supervised learning systems include quick inference, adaptability to complicated motions, and stable performance, but they have major drawbacks: They depend on large-scale annotated event datasets, which are expensive and laborious to collect due to the need for dense motion masks aligned with high-temporal resolution streams; ground-truth acquisition often requires auxiliary sensors or cameras that cannot match event sensors’ temporal granularity, resulting in misalignment and label noise; and they generalise poorly under domain shifts, especially distribution shifts.

Self-supervised and unsupervised learning approaches [53,54] recently emerged to lessen dependency on annotated event data. According to [53], EV-LayerSegNet is the first self-supervised encoder-decoder architecture designed for event-based motion segmentation. The network calculates affine optical flow and segmentation masks separately and fuses them via element-wise multiplication to create a motion-compensated flow map. This flow map deblurs the input event stream, with contrast maximization as the self-supervised learning goal. Wang et al. [54] developed Un-EvMoSeg, an unsupervised framework for segmenting IMOs from event streams without labels. The Geometric self-labeling method generates binary IMO pseudo-labels to supervise IMO segmentation networks.

### 2.2. Unsupervised Domain Adaptation for Event-Streams

Although unsupervised domain adaptation presents a possible remedy for a lack of labeled event data, its implementation in event-based vision is still limited. This is mainly attributable to the significant modality gap between asynchronous event streams and synchronous frame-based images, which obstructs direct representation-level alignment.

The preliminary methods utilized image-to-image translation [55] to facilitate cross-modal alignment through intermediate representations, enabling RGB-trained models to adapt to event inputs. For instance, Rebecq et al. [47] suggested the reconstruction of high-speed, HDR video from event streams to utilize its precise temporal resolution. Cadena et al. [56] enhanced E2VID by integrating spatially-adaptive denormalization (SPADE), which markedly improves the reconstruction quality of initial frames, while it incurs greater processing demands. Alternatively, methods such as VID2E [48,49] adopt a reverse translation strategy by generating synthetic event streams from RGB videos, enabling cross-modal representation learning through intermediate reconstruction spaces. However, reconstruction-based methods are limited by the requirement for paired training data, susceptible to artifacts due to the sparse and textureless characteristics of event streams, and hindered by domain discrepancies between synthetic and real-world data, ultimately limiting generalization to real-world settings. A better UDA strategy, EV-Transfer [50], introduces a systematic unsupervised domain adaptation technique for image-to-event transfer by decomposing the embedding space into motion-specific and modality-invariant elements. The framework employs adversarial learning to align the shared feature subspaces for tasks such as classification and detection. Nonetheless, it depends on modeling motion from static images to produce event representations, a fundamentally ill-posed technique that frequently compromises feature alignment crucial for cross-modal transfer. This limitation is resolved by ESS [43], which eliminates the necessity for motion hallucination by transferring tasks from static images to events using motion-invariant embeddings. These embeddings are generated using a pre-trained E2VID [47] encoder and aligned with single-image embeddings via a dedicated image encoder. However, hard alignment introduces artifacts that may impede the performance of subsequent tasks. Complementary to EV-Transfer, Gehrig et al. [45,57] suggest two contemporary methods that utilize contrastive and self-supervised learning to bridge the domain gap in a label-free adaptation context. They focus on learning domain-invariant features and show strong generalization on classification benchmarks.

Recent research has progressed Unsupervised Domain Adaptation (UDA) for event-based semantic segmentation through the investigation of pseudo-labeling and soft alignment methodologies. HPL-ESS [58] introduces a hybrid pseudo-labeling framework that combines spatial supervision from RGB-based models with temporal signals derived from event consistency, hence improving adaptation without requiring event labels. Simultaneously, CMESS [59] introduces a cross-modal learning methodology that employs attention-driven soft alignment. This methodology adaptively transmits semantic knowledge from labeled RGB frames to unlabelled event streams without relying on pixel-wise correspondences. Despite the fact that both methods reduce the reliance on motion hallucination and paired data, they are still reliant on intermediate RGB-based supervision and may encounter limitations in environments that are inadequately structured or extremely dynamic. The study in [60] introduces an unsupervised source-free cross-modal domain adaptation system designed to adapt object recognition models trained on RGB frames to event data without utilizing source domain data throughout the adaptation process. This study uses a pipeline for event-to-frame reconstruction and information transfer.

Unlike previous approaches that depend on motion hallucination, synthetic event generation, or intermediate RGB-based supervision, our method presents a systematic cross-modality UDA framework that conveys motion segmentation knowledge from labeled RGB-flow data to unlabelled event streams without necessitating paired data or scene-level alignment. Our method effectively aligns diverse sensor modalities by utilizing disentangled dual-path encoders for motion and appearance while ensuring consistency across several levels. Moreover, a streamlined inference pathway guarantees real-time implementation while preserving elevated segmentation precision in intricate, dynamic settings.

## 3. Preliminaries

This section provides a formal mathematical characterization of our proposed UDA system, aligned with the architectural block diagram illustrated below. It delineates the fundamental computational elements: feature extraction using dual encoders, pseudo-modality reconstruction for event data, domain alignment through shared representations, and the ultimate loss formulation.

Specifically, We separately extract appearance and motion information from RGB images and optical flow through distinct encoders, which we subsequently integrate to form a unified representation:

(1) $$F_{s}=F_{\mathrm{fuse}}\left(E_{\mathrm{img}}\left(I_{S}\left(i\right)\right),\;E_{\mathrm{flow}}\left(\mathrm{opt}\left(I_{S}\left(i\right),I_{S}\left(i-1\right)\right)\right)\right)$$

Here, $E_{\mathrm{img}}$ and $E_{\mathrm{flow}}$ denote the encoders for image and flow, respectively. $F_{\mathrm{fuse}}$ is a fusion module that combines both streams into a unified feature representation $F_{s}$.

### 3.1. Pseudo-Modality Reconstruction from Events

Given an input event stream *E*, we extract motion and appearance features separately using two dedicated encoders:

(2) $$z_{T}^{\mathrm{flow}}=E_{\mathrm{flow}}\left(E\right),\;\;\;z_{T}^{\mathrm{img}}=E_{\mathrm{img}}\left(E\right),$$

where $E_{\mathrm{flow}}$ and $E_{\mathrm{img}}$ denote the motion and appearance encoders, respectively. These features are then decoded into pseudo-modalities via two decoders:

(3) $${\hat{F}}_{T}=D_{\mathrm{flow}}\left(z_{T}^{\mathrm{flow}}\right),\;\;\;{\hat{I}}_{T}=D_{\mathrm{img}}\left(z_{T}^{\mathrm{img}}\right),$$

where ${\hat{F}}_{T}$ and ${\hat{I}}_{T}$ are pseudo-flow and pseudo-RGB representations, respectively. These outputs function as intermediate modalities for alignment with RGB and flow features from the source domain.

### 3.2. Feature Extraction in the Target Domain

Given the pseudo-image ${\hat{I}}_{T}$ and pseudo-flow ${\hat{F}}_{T}$ reconstructed from the target event stream *E*, we derive semantic and motion features through the RGB and flow encoders $f_{\mathrm{img}}$ and $f_{\mathrm{flow}}$, which were initially trained on source domain data.

(4) $${\hat{z}}_{T}^{\mathrm{img}}=f_{\mathrm{img}}\left({\hat{I}}_{T}\right),\;\;\;{\hat{z}}_{T}^{\mathrm{flow}}=f_{\mathrm{flow}}\left({\hat{F}}_{T}\right)$$

In this context, $f_{\mathrm{img}}$ and $f_{\mathrm{flow}}$ denote the appearance and motion encoders that were first trained on source RGB and optical flow inputs, respectively. The resultant feature maps ${\hat{z}}_{T}^{\mathrm{img}}$ and ${\hat{z}}_{T}^{\mathrm{flow}}$ are subsequently amalgamated to create a cohesive representation appropriate for domain alignment and task prediction:

(5) $$F_{R}=F_{\mathrm{fuse}}\left({\hat{z}}_{T}^{\mathrm{img}},{\hat{z}}_{T}^{\mathrm{flow}}\right)$$

### 3.3. Consistency Loss and Final Objective

To reconcile the cross-modality disparity between RGB-flow and event representations, our approach implements a series of multi-level consistency losses that facilitate the alignment process across the subsequent levels:

#### 3.3.1. Feature-Level Consistency

To enforce feature-level alignment, we implement two consistency losses that reduce the divergence between the appearance and motion features derived from the event data and those re-encoded from the reconstructed pseudo-modalities.

(6) $$\begin{array}{cc} L_{\mathrm{Cons}.\mathrm{Emb}}^{\mathrm{app}} & ={∥Z_{\mathrm{event}}^{\mathrm{app}}-{\hat{Z}}_{\mathrm{Rcs}.\mathrm{Img}}^{\mathrm{app}}∥}_{2} \end{array}$$

(7) $$\begin{array}{cc} L_{\mathrm{Cons}.\mathrm{Emb}}^{\mathrm{flow}} & ={∥Z_{\mathrm{event}}^{\mathrm{flow}}-{\hat{Z}}_{\mathrm{Rcs}.\mathrm{Img}}^{\mathrm{flow}}∥}_{2} \end{array}$$

#### 3.3.2. Prediction-Level Consistency

We enforce similarity between task predictions from both fused paths using Kullback–Leibler divergence:

(8) $$L_{\mathrm{pred}}=D_{\mathrm{KL}}\left(P_{T}∥P_{R}\right),$$

where $P_{T}$ and $P_{R}$ denote the predicted probability maps from the target features and reconstructed pseudo-modalities, respectively.

#### 3.3.3. Output-Level Consistency

We further align the final segmentation outputs through an $\ell_{2}$ loss between the two prediction branches:

(9) $$L_{\mathrm{out}}={∥S_{T}-S_{R}∥}_{2},$$

where $S_{T}$ and $S_{R}$ denote the soft segmentation scores from the event-driven and reconstruction-driven paths.

#### 3.3.4. Final Consistency Loss

The overall consistency objective is expressed as a weighted summation of the three levels:

(10) $$L_{\mathrm{consistency}}=\alpha L_{\mathrm{feat}}+\beta L_{\mathrm{pred}}+\gamma L_{\mathrm{out}},$$

where α, β, and γ are hyperparameters that regulate the impact of each consistency component.

## 4. Proposed Method

**Figure 2:**
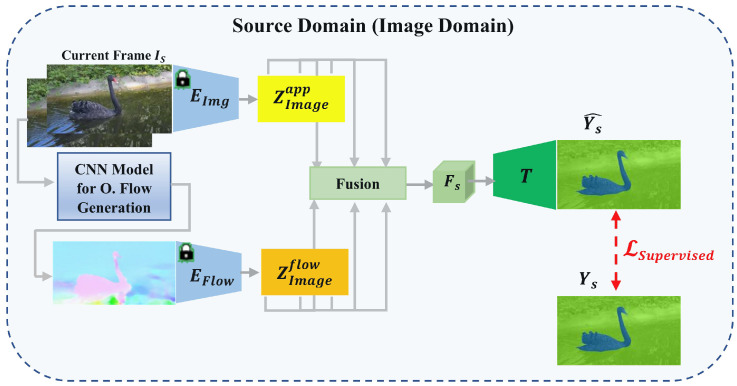
Supervised training pipeline in the source domain using RGB frames and optical flow for joint feature learning.

**Figure 3:**
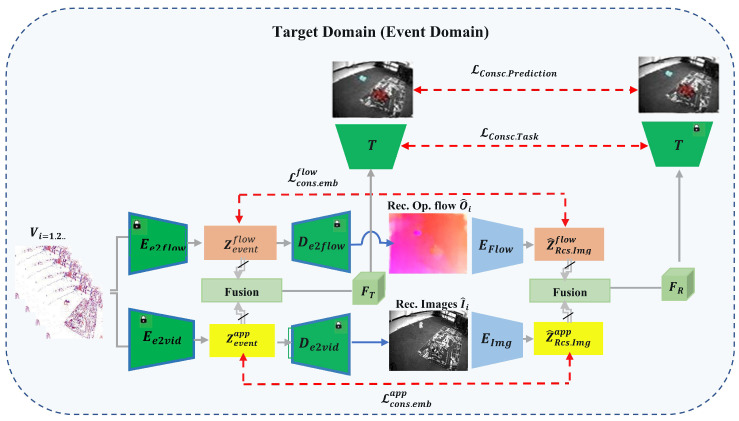
Unsupervised training pipeline in the target domain using event-based data, reconstruction modules, and consistency losses to learn domain-invariant representations.

This section presents our proposed cross-modal training paradigm for motion segmentation, facilitating efficient knowledge transfer from labelled RGB-flow data in the source domain to unlabelled event streams in the destination domain. The framework functions in an unsupervised and unpaired context, without requiring target labels or paired samples across domains. Our approach combines supervised learning in the source domain with unsupervised, consistency-based adaptation in the target domain. This joint training system enables the acquisition of domain-invariant representations, improving resilient motion segmentation across both domains. The comprehensive pipeline, illustrated in Figure 2 and  Figure 3, consists of three phases: (i) supervised dual-stream representation learning in the source domain, (ii) cross-modal reconstruction in the target domain, and (iii) multi-level cross-domain alignment via consistency constraints. Each phase in the framework fulfills a distinct role in enabling effective domain transfer.

In the source domain phase, every phase in the framework serves a specific function in facilitating efficient domain transfer. In the source domain phase, we train dual-stream encoders to separate appearance and motion information from RGB images and optical flow, respectively. The collected features are amalgamated and input into a common decoder, guided by ground-truth motion masks. This oversight directs the model to acquire complementary, modality-specific representations that are crucial for effective cross-domain generalization. During the second phase, voxelized event streams from the target domain are employed to reconstruct pseudo-RGB and pseudo-flow modalities using lightweight decoders. The pseudo-modalities are then processed through the frozen encoders, which are pre-trained on source RGB-flow data, yielding re-encoded features situated in the source-aligned representation space. To address the modality gap and maintain temporal consistency, we implement multi-level consistency constraints encompassing feature embeddings, task predictions, and output segmentation between the source and target domains. This allows the model to obtain strong, domain-independent, and temporally consistent representations for event-based motion segmentation without the need for target labels or paired samples. In the third phase, we implement multi-level cross-domain consistency to reconcile the representation disparity between the source and target modalities. Consistency constraints are implemented at three semantic tiers: (i) feature-level, aligning embeddings from source and reconstructed pseudo-modalities; (ii) prediction-level, correlating intermediate classifier outputs; and (iii) output-level, ensuring concordance on final segmentation masks. These objectives jointly guarantee that the model acquires temporally coherent, semantically aligned, and domain-invariant representations, even without paired or labeled target data. Unlike prior approaches that rely on paired supervision or motion hallucination, our method aligns reconstructed pseudo-modalities with RGB-flow features through a unified and lightweight adaptation strategy, enabling scalable and accurate motion segmentation in real-world scenarios.

### 4.1. Source Domain Training (RGB Video)

In the source domain, we assume access to labeled RGB videos, where each frame $I_{S}\left(i\right)$ is associated with a ground truth label $Y_{S}\left(i\right)$. The aim is to train a motion segmentation model $T_{\mathrm{SD}}$ using RGB-flow inputs under full supervision, allowing both the dual-stream feature encoders and the task-specific decoder to be effectively tuned to the target domain.

**Feature Extraction and Fusion:**

Figure 2 depicts a dual-branch encoder architecture that independently extracts appearance and motion data, which are then integrated and decoded into segmentation masks. The two modalities yield complementary information: the appearance stream encodes spatial textures and object boundaries, whereas the motion stream collects temporal dynamics and movement patterns. This difference is important for solving specific problems related to each type of data motion information, which helps clear up appearance issues caused by lighting or occlusion, while appearance information facilitates understanding of motion when it is weak or slow. The resultant joint representation provides a strong and distinctive foundation for precise motion segmentation.

Formally, for a sequential frames $I_{S}\left(i\right)$ and $I_{S}\left(i-1\right)$, the appearance and motion features are extracted as follows:

(11) $$\begin{array}{cc} Z_{\mathrm{Image}}^{\mathrm{app}}\left(k\right) & =E_{\mathrm{img}}\left(I_{S}\left(i\right)\right) \end{array}$$

(12) $$\begin{array}{cc} Z_{\mathrm{Image}}^{\mathrm{flow}}\left(k\right) & =E_{\mathrm{flow}}\left(\mathrm{opt}\left(I_{S}\left(i\right),I_{S}\left(i-1\right)\right)\right) \end{array}$$

where opt(·,·) represents the operation of computing optical flow between two consecutive frames. Our approach utilizes a well-trained RAFT [61] model to produce high-quality optical flow maps from sequential RGB frames.

The extracted features are then fused to form a joint representation:

(13) $$F_{S}=\Phi \left(Z_{\mathrm{Image}}^{\mathrm{app}},Z_{\mathrm{Image}}^{\mathrm{flow}}\right)$$

The integrated representation is then transmitted to the task decoder for supervised segmentation.

**Supervised Classification:** A classifier T(·) is trained using the fused features $F_{S}$ with cross-entropy loss:

(14) $$\begin{array}{cc} {\hat{Y}}_{S} & =T(F_{S}) \end{array}$$

(15) $$\begin{array}{cc} L_{\mathrm{Supervised}} & =-\frac{1}{C}\sum_{i=1}^{C}y_{i}\log\left({\hat{Y}}_{S}\left(i\right)\right) \end{array}$$

To enhance representation learning from RGB frames, we initialize the appearance and motion encoders, $E_{\mathrm{img}}$ and $E_{\mathrm{flow}}$, using pretrained weights from the Isomerous Transformer [62], an advanced architecture initially developed for zero-shot video object segmentation. Utilizing these pretrained weights allows the encoders to more effectively capture spatial textures and temporal dynamics, therefore expediting convergence and enhancing segmentation performance in the source domain.

### 4.2. Target Domain Adaptation (Event Streams)

Unlike the source domain, the target domain consists of unlabelled, asynchronous event streams that are devoid of dense pixel-level annotations and display sparse spatio-temporal organization. Our aim is to align features obtained from event streams with those from the source RGB-flow domain by utilizing unsupervised supervisory signals. The adaptation process occurs in three sequential stages: (i) extracting semantic and motion features from voxelized event representations, (ii) reconstructing pseudo-RGB and pseudo-flow modalities from event features, and (iii) enforcing multi-level consistency constraints to ensure alignment in feature space, prediction space, and output space (Figure 3).

**(1) Event-Based Feature Extraction:** To manage the sparse and asynchronous characteristics of event data, the input event stream is initially aggregated into a voxel grid representation, denoted as $v_{i}$, which compactly encodes the spatio-temporal event patterns over a predefined temporal window. Two pretrained encoders are utilised to extract complimentary features: $E_{e2vid}$ reconstructs appearance information resembling to RGB frames, while $E_{e2flow}$ is intended to extract motion dynamics similar to optical flow. From each voxel grid ($v_{i}$), we derive modality-specific embeddings:

(16) $$\begin{array}{cc} Z_{\mathrm{event}}^{\mathrm{app}}\left(k\right) & =E_{e2vid}\left(v_{i}\right) \end{array}$$

(17) $$\begin{array}{cc} Z_{\mathrm{event}}^{\mathrm{flow}}\left(k\right) & =E_{e2flow}\left(v_{i}\right) \end{array}$$

The two feature streams are subsequently fused using a modality integration function to derive a joint representation:

(18) $$F_{T}=\Psi \left(Z_{\mathrm{event}}^{\mathrm{app}},Z_{\mathrm{event}}^{\mathrm{flow}}\right)$$

This integrated representation functions as the semantic foundation for cross-modal reconstruction and consistency alignment with the source domain in subsequent stages.

**(2) Cross-Modal Reconstruction:** To bridge the modality gap between the event-based and RGB-flow domains, the retrieved event features are transformed into pseudo-frame modalities. Particularly, appearance and motion characteristics derived from $E_{e2vid}$ and $E_{e2flow}$ are processed by pretrained decoders to generate pseudo-RGB images $\hat{I}i$ and pseudo-flow maps $\hat{F}i$:

(19) $$\begin{array}{cc} {\hat{I}}_{i} & =D_{e2vid}\left(Z_{\mathrm{event}}^{\mathrm{app}}\right) \end{array}$$

(20) $$\begin{array}{cc} {\hat{F}}_{i} & =D_{e2flow}\left(Z_{\mathrm{event}}^{\mathrm{flow}}\right) \end{array}$$

These synthetic modalities function as a bridge to utilise the representational power of source-trained encoders. The recovered RGB and flow outputs are re-encoded with the frozen feature extractors $E_{\mathrm{img}}$ and $E_{\mathrm{flow}}$, which were previously trained on labelled RGB-flow data, to provide intermediate representations:

(21) $$\begin{array}{cc} {\hat{Z}}_{\mathrm{Rcs}.\mathrm{Img}}^{\mathrm{app}} & =E_{\mathrm{img}}\left({\hat{I}}_{i}\right) \end{array}$$

(22) $$\begin{array}{cc} {\hat{Z}}_{\mathrm{Rcs}.\mathrm{Img}}^{\mathrm{flow}} & =E_{\mathrm{flow}}\left({\hat{F}}_{i}\right) \end{array}$$

The re-encoding process integrates the reconstructed pseudo-modalities into the same representational space as the source domain, facilitating alignment among modalities. By maintaining spatial and temporal within this shared space, the approach enhances domain-invariant representation learning, thus enabling robust generalization without paired supervision.

**(3) Consistency Losses:** To effectively close the modality gap and promote strong cross-domain adaptation, we utilise a multi-level consistency technique that ensures alignment between event-derived characteristics and their source domain equivalents. This consistency is enforced at three semantic levels: feature space, prediction space, and output space. We define the following consistency losses: Feature-Level Consistency ensures that latent representations from source and target domains occupy similar embedding spaces:

(23) $$\begin{array}{cc} L_{\mathrm{Cons}.\mathrm{Emb}}^{\mathrm{app}} & ={∥Z_{\mathrm{event}}^{\mathrm{app}}-{\hat{Z}}_{\mathrm{Rcs}.\mathrm{Img}}^{\mathrm{app}}∥}_{2} \end{array}$$

(24) $$\begin{array}{cc} L_{\mathrm{Cons}.\mathrm{Emb}}^{\mathrm{flow}} & ={∥Z_{\mathrm{event}}^{\mathrm{flow}}-{\hat{Z}}_{\mathrm{Rcs}.\mathrm{Img}}^{\mathrm{flow}}∥}_{2} \end{array}$$

To enable higher-level consistency objectives, the fused event representation $F_{T}$ and the re-encoded features $F_{R}$ obtained from reconstructed pseudo-modalities are both forwarded through the task network T(·), allowing us to compute consistency not only at the feature level but also at the prediction and output levels. Prediction-Level Consistency reduces the disparity between class probability outputs from both domains:

(25) $$L_{\mathrm{Cons}.\mathrm{Prediction}}=D_{KL}\left(T\left(F_{T}\right)\|\;T\left(F_{R}\right)\right)+D_{KL}\left(T\left(F_{R}\right)\|\;T\left(F_{T}\right)\right)$$

Output-Level Consistency promotes the stability of segmentation masks across several domains, hence maintaining alignment at the task level:

(26) $$L_{\mathrm{Cons}.\mathrm{Task}}=\sum_{k}{∥T^{k}\left(F_{T}\right)-T^{k}\left(F_{R}\right)∥}_{2}$$

**(4) Total Loss Function:** To train the entire framework, we define a joint objective that balances the supervised loss in the source domain with the unsupervised alignment losses in the target domain. Each term plays a distinct role:

- $L_{\mathrm{Supervised}}$: Ensures discriminative learning on source data.
- $L_{\mathrm{Cons}.\mathrm{Emb}}^{\mathrm{flow}},L_{\mathrm{Cons}.\mathrm{Emb}}^{\mathrm{app}}$: Align low-level ap and motion features.
- $L_{\mathrm{Cons}.\mathrm{Task}}$: Enforces similarity between fused embeddings from both event and reconstruction paths.
- $L_{\mathrm{Cons}.\mathrm{Prediction}}$: Regularizes classifier outputs for consistency across modalities.

Importantly, the combination of embedding-level losses ($L_{\mathrm{Cons}.\mathrm{Emb}}^{\mathrm{app}},L_{\mathrm{Cons}.\mathrm{Emb}}^{\mathrm{flow}}$) and task or prediction-level consistency losses ($L_{\mathrm{Cons}.\mathrm{Task}},L_{\mathrm{Cons}.\mathrm{Prediction}}$) enforces that the representations learned from event-based inputs are semantically aligned with those from RGB videos. As a result, the fused event representation $F_{T}$ becomes domain-invariant, allowing the classifier trained on source domain data to generalize to target domain data without needing labeled target samples.

The final loss is computed as follows:

(27) $$L_{\mathrm{Total}}=\lambda_{0}L_{\mathrm{Supervised}}+\lambda_{1}L_{\mathrm{Cons}.\mathrm{Emb}}^{\mathrm{flow}}+\lambda_{2}L_{\mathrm{Cons}.\mathrm{Emb}}^{\mathrm{app}}+\lambda_{3}L_{\mathrm{Cons}.\mathrm{Task}}+\lambda_{4}L_{.\mathrm{Prediction}}$$

As a way to ensure reproducibility, we summarize all the steps described above into a unified Algorithm 1, encompassing both source-domain supervision and target-domain adaptation. The pseudocode captures the key aspects of feature extraction, cross-modal reconstruction, and multi-level consistency optimization.
**Algorithm 1** Unsupervised Domain Adaptation via Consistency-Based Cross-Modal TrainingInitialize all networks: $E_{\mathrm{img}}$, $E_{\mathrm{flow}}$, *T*, $E_{e2vid}$, $E_{e2flow}$, $D_{e2vid}$, $D_{e2flow}$
**for** each training iteration **do** Sample labeled source batch $\left\{I_{S},Y_{S}\right\}$ Extract $Z_{\mathrm{Image}}^{\mathrm{app}},Z_{\mathrm{Image}}^{\mathrm{flow}}\to F_{S}\to {\hat{Y}}_{S}$▹ Used in Equations (11)–(13) Compute $L_{\mathrm{Supervised}}$▹ Equation (15) Sample voxel grid representation batch $\left\{v_{i}\right\}$ Extract $Z_{\mathrm{event}}^{\mathrm{app}},Z_{\mathrm{event}}^{\mathrm{flow}}\to F_{T}\to {\hat{Y}}_{T}$▹ Used in Equations (16) and (17) Reconstruct ${\hat{I}}_{i},{\hat{F}}_{i}$ from decoders ▹ Used in Equations (19) and (20) Extract ${\hat{Z}}_{\mathrm{Rcs}.\mathrm{Img}}^{\mathrm{app}},{\hat{Z}}_{\mathrm{Rcs}.\mathrm{Img}}^{\mathrm{flow}}\to F_{R}\to {\hat{Y}}_{R}$▹ Used in Equations (21) and (22) Compute all consistency losses: $L_{\mathrm{Cons}.\mathrm{Emb}}^{\mathrm{app}},L_{\mathrm{Cons}.\mathrm{Emb}}^{\mathrm{flow}},L_{\mathrm{Cons}.\mathrm{Task}},L_{\mathrm{Cons}.\mathrm{Prediction}}$ Compute total loss $L_{\mathrm{Total}}$▹ Equation (27) Backpropagation and update parameters**end for**

Here we provide a step-by-step explanation of the training procedure presented in the pseudocode.

1. Initialize all components of the model. Pretrained encoders and decoders are frozen during adaptation.
2. Begin the training loop over source and target data.
3. Sample a mini-batch of labeled RGB frames and their labels from the source domain.
4. Extract appearance and flow features using $E_{\mathrm{img}}$ and $E_{\mathrm{flow}}$, then fuse them to compute $F_{S}$. Predict labels ${\hat{Y}}_{S}$.
5. Compute supervised classification loss $L_{\mathrm{Supervised}}$ using Equation (15).
6. Sample an voxel grid representation of the event data from the target domain.
7. Extract appearance and motion features from events, fuse them, and generate the initial prediction ${\hat{Y}}_{T}$.
8. Reconstruct synthetic RGB image ${\hat{I}}_{i}$ and optical flow ${\hat{F}}_{i}$ from event features.
9. Extract appearance and flow features again using RGB-trained encoders, generate $F_{R}$ and prediction ${\hat{Y}}_{R}$.
10. Compute all consistency losses between the event and reconstructed paths.
11. Combine all losses into a total loss $L_{\mathrm{Total}}$ using Equation (27).
12. Perform backpropagation to update trainable parameters.

### 4.3. Inference Phase (Run-Time Deployment)

During inference, the model uses only the efficient and lightweight event-driven pipeline for real-time motion segmentation. Specifically, the input voxel grid representation of the event stream $v_{i}$ s passed through the appearance encoder $E_{e2vid}$ and the motion encoder $E_{e2flow}$ to derive semantic features $Z_{\mathrm{event}}^{\mathrm{app}}$ and flow $Z_{\mathrm{event}}^{\mathrm{flow}}$, respectively. These features are then fused and passed to the classifier *T* to get the final prediction ${\hat{Y}}_{T}$.

**Figure 4:**
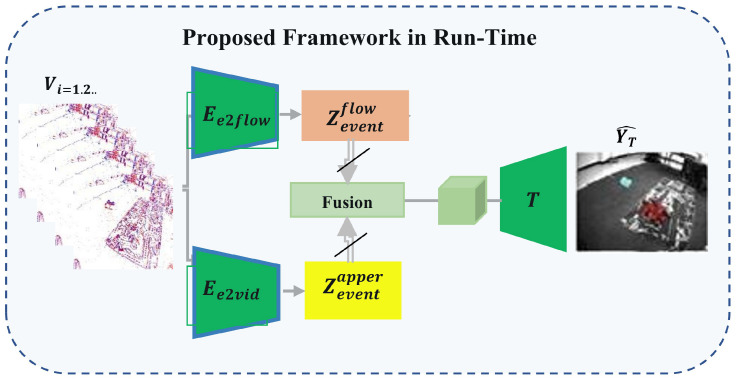
The proposed lightweight inference pipeline used at run-time for event-based input.

As depicted in Figure 4, the inference phase utilizes solely the event-based encoders and the trained classifier, hence obviating the necessity for reconstruction or domain alignment modules. This architecture guarantees efficient, low-latency implementation appropriate for practical applications.

## 5. Experimental Results

### 5.1. Benchmarks Dataset

We evaluate our approach using two complementary benchmarks tailored for event-based motion segmentation: EVIMO2 [63] and MOD++ [27]. These datasets collectively offer a varied and organized framework for evaluating model resilience and generalization in both real-world and synthetic contexts.

**Figure 5:**
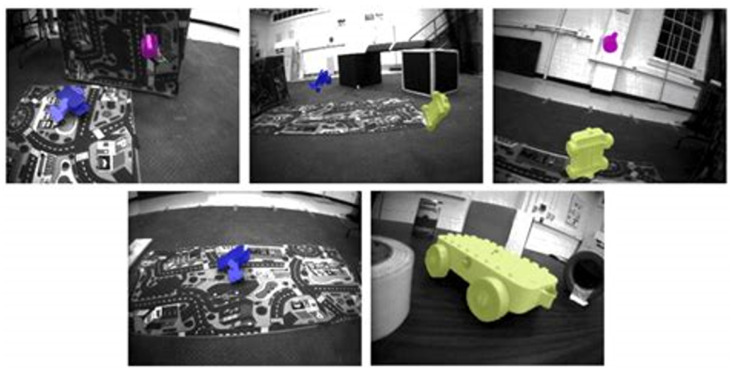
Indoor corridor scene from EVIMO2 with converging walls and textured flooring, representing a complex geometric background for evaluating motion segmentation in event-based vision systems.

**EVIMO2** comprises authentic indoor environments recorded by a high-resolution DAVIS camera (480 × 640), providing temporally synchronized event streams and RGB frames, as well as comprehensive ground truth for depth, optical flow, and per-frame motion segmentation. The dataset comprises more than 34 sequences (24 designated for training and 10 for testing), encompassing a diverse array of object sizes and motion velocities, ranging from small, rapidly moving entities to large, slowly moving ones, across varying lighting conditions, including low-light and high-dynamic-range environments. The aforementioned problematic features render EVIMO2 especially adept at assessing segmentation performance on sparse and asynchronous input data. Figure 5 demonstrates that the dataset encompasses diverse background geometries (e.g., hallways, cluttered rooms, textured surfaces), intended to evaluate the model’s capacity to differentiate foreground motion from parallax or ego-motion.

**MOD++** is a synthetic benchmark designed for event-based motion comprehension in highly dynamic settings. The collection consists of 42 sequences generated with a simulated DAVIS240C camera, illustrating intricate interactions among various independently moving objects (IMOs), encompassing collisions and explosions. MOD++ offers high-frequency (1 kHz) ground-truth annotations for flow, depth, and segmentation masks. The regulated composition of scenes and accurate labels render it optimal for detailed temporal study of segmentation efficacy amid organized motion patterns and object interactions. For example, Figure 5 illustrates various types of background geometries present in the EVIMO2 dataset, which is crucial for training and evaluating motion segmentation models using event cameras. The image displays several scenes with distinct background structures such as: flat walls, corridors, rooms with clutter, furniture, posters, or textured surfaces. Each scene has independently moved objects placed in front of these backgrounds. The objective is to test the model’s capacity to differentiate object motion from background parallax or camera-induced motion.

Training in the source domain utilized labeled RGB images alongside their associated optical flow maps from the DAVIS-16 dataset [64], allowing the dual-stream segmentation model to acquire motion and appearance representations under complete supervision.

To quantify the domain shift between the source and target domains, we computed the Fréchet Distance between the latent feature distributions extracted from the source (RGB–flow) and target (event) domains using the shared fusion backbone. The cross-domain Fréchet Distance between EVIMO2 and MOD++ feature embeddings was 78.4, compared to 29.7 for intra-domain samples, confirming a strong modality and distributional gap. Regarding label noise, MOD++ segmentation masks are semi-automatically generated from RGB keyframes via optical flow propagation, which can introduce spatial misalignments and boundary errors particularly for fast-moving or low-texture regions. We observed an estimated 3–5% label uncertainty in boundary pixels based on manual inspection of 50 randomly sampled frames. To address these issues, our proposed framework employs multi-level consistency losses ($L_{\mathrm{cons}.\mathrm{emb}}$, $L_{\mathrm{cons}.\mathrm{task}}$, $L_{\mathrm{cons}.\mathrm{pred}}$) that promote domain-invariant feature learning and improve robustness against noisy supervision.

### 5.2. Experimental Setup and Baseline Models Details

We performed experiments on the EVIMO2 and MOD++ datasets. All models were trained solely on synthetic event streams produced by the ESIM simulator [65], without any access to actual target labels or frame-wise correspondence. This ensures a fully unpaired unsupervised domain adaptation (UDA) setting, where synthetic and real domains remain completely disjoint throughout training. Input data consisted of voxelized event frames accumulated over fixed temporal windows. A lightweight U-Net architecture served as the segmentation backbone for all UDA methods. Training utilized the Adam optimizer with an initial learning rate, a cosine decay schedule, and standard augmentations including random flips, cropping, and event normalization. Each model underwent training for 100 epochs with a batch size of 8. For MOD++, which emphasizes monocular event-based motion segmentation, training was performed entirely on synthetic data, with evaluation on real sequences.

We evaluated several UDA strategies, including E2VID [47], EV-Transfer [50], and SHOT [66], as well as our proposed method, which includes dual-stream feature disentanglement and multi-level consistency alignment. As a further reference, a No-UDA baseline was given in which the model was trained solely on synthetic data and tested directly on the target domain with no adaptation. We also present a hybrid model, designated as Supervised+UDA, which integrates supervised training on labeled event data with the proposed UDA framework. This variation utilizes labeled RGB images from the source domain to enhance cross-domain alignment and augment segmentation performance in the target domain. This hybrid configuration acts as an upper limit to evaluate the impact of our domain adaptation modules under conditions of restricted supervision in the target domain. Performance was measured using metrics such as Mean Intersection over Union (mIoU), Pixel Accuracy, and F1-Score, which demonstrated each method’s capacity to generalize from synthetic to real-world event data across both datasets.

To ensure efficient training and evaluation of the proposed UDA model for motion segmentation, high-performance hardware was utilized. The training was performed on an NVIDIA Tesla V100 GPU (32 GB VRAM) in conjunction with an Intel Xeon Gold 6248 CPU (20 cores) and 256 GB RAM, facilitating extensive processing and efficient data management. Testing was conducted on a machine equipped with an NVIDIA RTX 3090 GPU (24 GB VRAM), an Intel Core i9-10900K CPU, 64 GB RAM, and a 1 TB SSD, facilitating real-time inference on high-resolution data in both simulated and real-world contexts.

### 5.3. Results for Motion Segmentation Using UDA Models

We evaluated several unsupervised domain adaptation (UDA) methods for motion segmentation on two event-based datasets: EVIMO2 and MOD++. These experiments aimed to assess the generalization ability of UDA models trained solely on synthetic data and tested directly on real-world sequences without any target supervision. Given the sparse, asynchronous, and high-temporal-resolution nature of event data, conventional models often fail to perform effectively without domain adaptation. To address this, we benchmarked four representative UDA methods E2VID, EV-Transfer, SHOT, and a Proposed UDA under consistent training settings. Performance was measured using mIoU, accuracy, and F1-score.

Across both datasets, all UDA models substantially outperformed the source-only baseline, confirming their ability to bridge the domain gap. The Proposed UDA consistently achieved the best results, particularly excelling in segmenting complex motion boundaries. SHOT and EV-Transfer also demonstrated strong performance, benefiting from temporal consistency and adversarial alignment. Although E2VID showed the lowest relative performance, it still improved over the non-adaptive baseline.

Unless otherwise specified, all results are reported as mean ± standard deviation over *k*-fold cross-validation. Specifically, the dataset is partitioned into k=5 non-overlapping folds, ensuring that each fold preserves the overall distribution of motion and illumination conditions. In each experiment, four folds are used for training and the remaining one for testing, rotating until all folds have served as the test set. The final score represents the average and standard deviation across the five folds. For each method, statistical significance with respect to the strongest baseline is assessed using a paired *t*-test over per-fold mIoU values (p<0.05), and significant improvements are marked with ^†^.

Table 1 presents the results of this experiment. Proposed UDA achieved the best performance with an mIoU of 63.4±0.8%, accuracy of 83.1±0.6%, and F1-score of 71.3±0.8%, outperforming the Source Only baseline (mIoU: 41.5±1.1%, F1: 47.3±1.2%). SHOT and EV-Transfer also showed strong improvements, reaching mIoUs of 60.2±0.9% and 58.9±0.8%, respectively. On the MOD++ dataset, which includes more complex multi-motion scenarios, the Proposed UDA again led with an mIoU of 56.8±0.7%, accuracy of 79.4±0.6%, and F1-score of 65.7±0.7%. SHOT and EV-Transfer followed closely, indicating their robustness in transferring knowledge to challenging real-world sequences. E2VID, while less competitive (mIoU: 48.7±0.8% on MOD++), still improved significantly over the Source Only baseline (mIoU: 42.3±1.0%, based on text description). These results confirm that integrating domain adaptation mechanisms, especially those combining spatial alignment and temporal consistency, can significantly enhance motion segmentation performance with event cameras in real-world environments.

### 5.4. Per-Sequence Analysis of UDA Models on Event-Based Benchmarks

We evaluated the sequence-level efficacy of unsupervised domain adaptation (UDA) methods for event-based motion segmentation by benchmarking four typical models, E2VID, EV-Transfer, SHOT, and the Proposed UDA, on the EVIMO2 and MOD++ datasets. These benchmarks present considerable hurdles owing to the synthetic-to-real domain transition, intricate motion dynamics, and varied lighting conditions.

Table 2 demonstrates that all UDA approaches consistently surpassed the Source Only baseline across sequences. The proposed UDA attained the highest overall accuracy in both standards. On EVIMO2, it attained an average mIoU of 62.8±1.1%, above SHOT (59.2±0.9%) and EV-Transfer (57.3±0.8%), whilst E2VID achieved 49.6±0.9%. The Source Only model exhibited markedly inferior performance, with a score of 39.8±0.9%. On MOD++, the proposed UDA exhibited exceptional performance across individual sequences, with a maximum mIoU of 58.1±0.7% on sequence 4 and an overall strong performance. SHOT and EV-Transfer exhibited competitive performance; however, E2VID was the least effective among UDA approaches, albeit it was still above the non-adaptive baseline.

The Proposed UDA presents a significant margin of improvement: +23.0% on EVIMO2 and up to +18.3% on MOD++ (e.g., from 39.8% to 58.1% on seq4). These results demonstrate the model’s strong adaptation ability across distinct event domains, even when trained exclusively on synthetic data. While current experiments are limited to indoor datasets, the observed improvements suggest promising generalization potential for more complex real-world scenarios, which will be further explored in future work.

The considerable performance gap relative to the Source Only baseline confirms the effectiveness of our multi-level consistency framework in obtaining robust, domain-invariant representations that address the distributional shift between synthetic and real event data. This validates the design choice to enforce alignment across embedding, prediction, and output levels to enable efficient knowledge transfer without target monitoring.

Among the assessed approaches, SHOT demonstrated consistently steady performance across sequences, highlighting the effectiveness of temporal consistency in managing real-world motion patterns. EV-Transfer exhibited robust outcomes by utilizing pixel and feature-level alignment to get competitive mid-range accuracy. Although E2VID exhibited the lowest performance among UDA models, it nonetheless demonstrated significant enhancements over the non-adaptive baseline, confirming that even basic adaptation procedures can produce measurable advantages. These results emphasize the importance of utilizing UDA techniques in event-based motion segmentation and stress the essential function of spatiotemporal alignment mechanisms for ensuring dependable implementation in intricate real-world settings.

### 5.5. Quantitative Analysis of Motion Segmentation Under UDA and Supervised Settings

**Quantitative and Visual Comparison of UDA Models:** To evaluate the cross-domain generalization capacities of different UDA approaches, we provide a comparative visual and quantitative analysis of motion segmentation performance on the EVIMO2 and MOD++ benchmarks. The assessed models comprise a non-adaptive Source Only baseline, E2VID, EV-Transfer, SHOT, and our Proposed UDA approach. Performance is measured using the mean Intersection over Union (mIoU) metric and illustrated by bar and line charts, which reflect both the average outcomes across datasets and the detailed performance across individual sequences.

**Figure 6:**
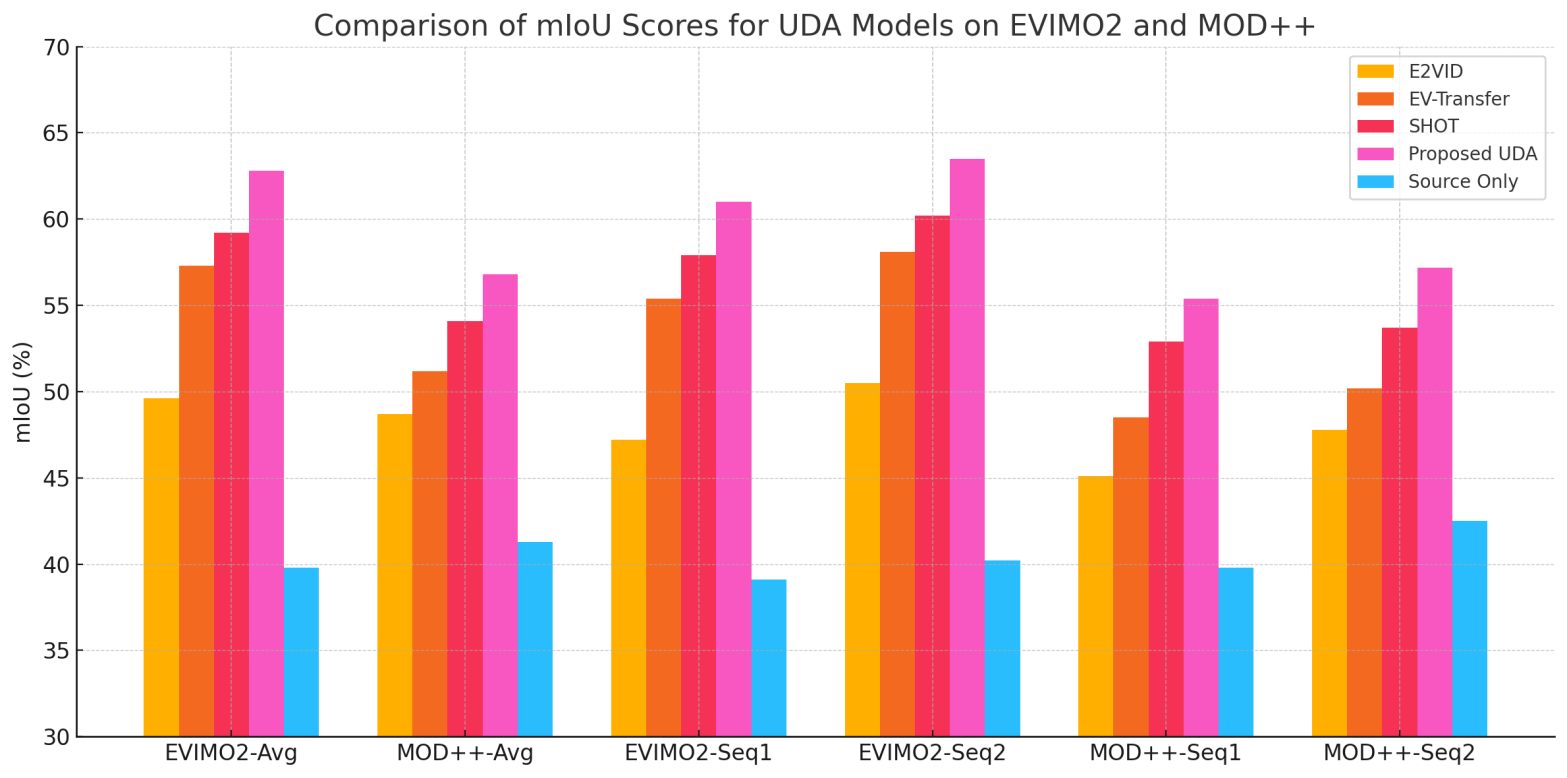
Comparison of mIoU scores for UDA models across EVIMO2 and MOD++ datasets and sequences.

Figure 6 demonstrates that all UDA approaches significantly exceed the Source Only baseline, confirming the essentiality of domain adaptation in transitioning from synthetic to real event data. The Proposed UDA consistently attains the greatest mIoU across both datasets, exhibiting significant robustness and generalization in demanding real-world scenarios. SHOT and EV-Transfer exhibit competitive performance, owing to their temporal consistency and pixel-level alignment techniques, respectively. Despite E2VID demonstrating comparatively inferior performance, it yet provides quantifiable enhancements over the baseline, suggesting that even rudimentary reconstruction-based methodologies can facilitate cross-domain transfer. These findings validate the efficacy of UDA approaches in reducing domain discrepancies and enhancing segmentation precision for event-based motion analysis.

**Figure 7:**
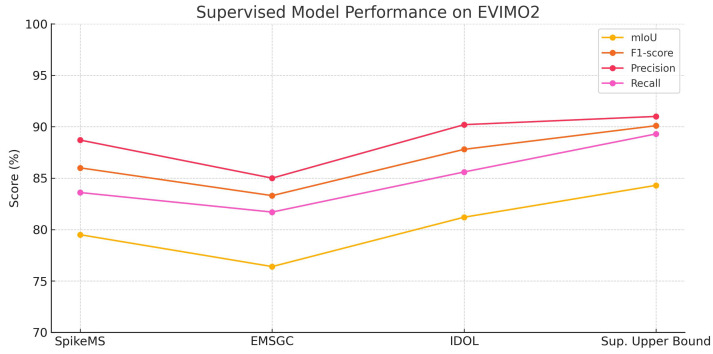
Supervised model performance on EVIMO2 dataset.

**Figure 8:**
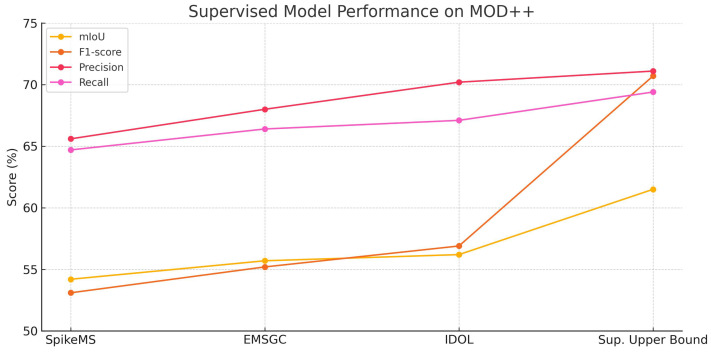
Supervised model performance on MOD++ dataset.

**Quantitative Evaluation of Supervised Models.**  Figure 7 and Figure 8 illustrate the performance of four supervised models across four assessment metrics: mIoU, F1-score, Precision, and Recall. In both datasets, the supervised upper bound attains the greatest scores. IDOL demonstrates exceptional performance, notably in precision and recall. SpikeMS has robust performance on EVIMO2 but experiences considerable degradation on MOD++, while EMSGC sustains stable, albeit marginally inferior, performance. These charts function as standards for assessing the comparative efficacy of UDA approaches.

### 5.6. Reconstruction Fidelity Analysis

To assess the reliability of the reconstructed pseudo-modalities used for domain alignment, we conduct a quantitative and qualitative analysis of the pseudo-RGB and pseudo-flow representations generated from sparse event streams. These reconstructed modalities (${\hat{I}}_{i}$ and ${\hat{O}}_{i}$) serve as the intermediate bridge between the unlabeled event domain and the labeled RGB–flow source domain. Evaluating their fidelity is crucial to ensure that the subsequent feature- and prediction-level consistency losses are meaningful rather than biased by reconstruction artifacts. Using the EVIMO2 dataset, which provides corresponding grayscale intensity and optical flow ground truth, we compute three standard metrics: (i) the *Peak Signal-to-Noise Ratio (PSNR)* and (ii) the *Structural Similarity Index (SSIM)* for pseudo-RGB reconstruction quality, and (iii) the *End-Point Error (EPE)* for pseudo-flow estimation accuracy. The results in Table 3 indicate that the reconstructed pseudo-RGB frames achieve a PSNR of 27.8 dB and SSIM of 0.84, while the pseudo-flow reconstruction obtains an EPE of 0.43. These values demonstrate that both pseudo-modalities maintain sufficient structural and motion fidelity to support reliable cross-modal alignment. For datasets such as MOD++, where ground-truth intensity and flow are not available, we report the results only on EVIMO2.

**Figure 9:**
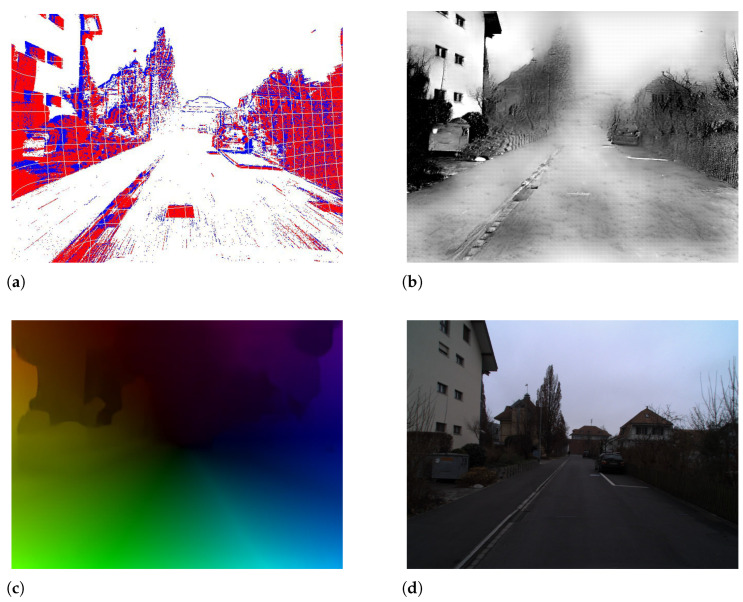
Qualitative examples of pseudo-RGB and pseudo-flow reconstruction from event data. **Top row**: (**a**) input event frame, (**b**) reconstructed pseudo-RGB. **Bottom row**: (**c**) reconstructed pseudo-flow, (**d**) corresponding ground truth. The reconstructions preserve semantic structure and motion boundaries suitable for downstream segmentation.

To further assess the influence of reconstruction fidelity on downstream segmentation, we remove the reconstruction consistency losses ($L_{\mathrm{Cons}.\mathrm{Emb}}$ and $L_{\mathrm{Cons}.\mathrm{Prediction}}$) from the adaptation stage. As summarized in Table 4, this modification results in a 3.1 mIoU decrease on EVIMO2 and 2.6 mIoU on MOD++, confirming that even with mild reconstruction noise, the pseudo-modalities provide valuable cross-domain supervision. Figure 9 illustrates visual examples of reconstructed pseudo-RGB and pseudo-flow frames compared with their ground-truth counterparts. Despite the inherent sparsity of event data, the reconstructions preserve fine spatial details and motion boundaries, providing an effective bridge between the event and RGB-flow domains. Overall, these analyses confirm that the reconstruction process yields sufficiently accurate pseudo-modalities for multi-level consistency learning, thereby supporting robust unsupervised domain adaptation without requiring paired RGB-event data.

### 5.7. Qualitative Analysis of Model Outputs

To augment the quantitative results, we present a comprehensive qualitative assessment of motion segmentation outcomes in both unsupervised domain adaptation and supervised contexts. This comparison elucidates the visual strengths and constraints of each model in processing complex event-based sequences from EVIMO2 and MOD++.

**Figure 10:**
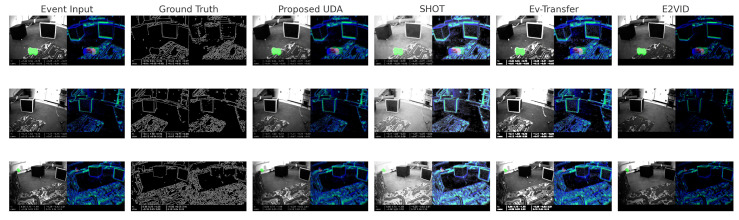
Qualitative comparison of motion segmentation outputs from different UDA models on EVIMO2 sequence.

Figure 10 presents a qualitative comparison of motion segmentation outputs using four UDA models, E2VID, EV-Transfer, SHOT, and the Proposed UDA models, on three selected frames from the EVIMO2 sequence ’seq-00’. Each row contains the RGB event input, simulated edge-based ground truth, and associated predictions from proposed UDA, SHOT, EV-Transfer, and E2VID. The Proposed UDA predictions appear smooth and consistent with prominent object boundaries, demonstrating their robustness in domain transfer. SHOT improves contrast in dynamic regions and aligns effectively with object contours. EV-Transfer introduces structural dilation effects, which help capture larger moving areas but may blur fine details. E2VID tends to erode edges, possibly under-segmenting motion areas. Overall, this visual layout helps assess how each model interprets motion dynamics in event-based input, offering a practical, side-by-side evaluation of their qualitative performance.

**Figure 11:**
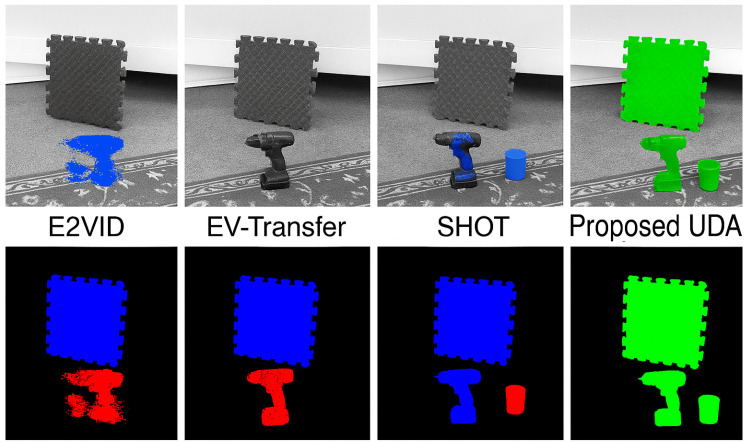
Qualitative comparison of motion segmentation outputs from different UDA models on MOD++ sequence.

Figure 11 presents a similar qualitative comparison on the MOD++ dataset. The layout is arranged in a 2 × 4 grid, with the top row displaying grayscale reconstructions overlaid with color-coded motion segmentation masks and the bottom row showing binary segmentation maps for each respective model. In the top row, the colored overlays highlight the motion segmentation boundaries generated by each model. These overlays reveal how effectively each approach distinguishes independently moving objects, such as the power drill, foam mat, and cup, within a cluttered indoor environment. The E2VID output shows limited boundary sharpness and noisy overlays, reflecting its weaker performance in directly leveraging event streams for segmentation. EV-Transfer improves on this by applying adversarial adaptation, resulting in clearer, though occasionally fragmented, segmentation. SHOT further enhances spatial consistency by refining predictions using self-training and entropy regularization, providing cleaner contours. The Proposed UDA model, however, delivers the most accurate and refined results, with precise segmentation masks, minimal background interference, and clear separation between distinct objects in motion. The binary masks in the bottom row reinforce these observations by isolating object silhouettes based on motion clusters. The Proposed UDA model produces clean, coherent shapes with strong alignment to object boundaries, while the other methods display varying degrees of over-segmentation, under-segmentation, or background leakage. This figure effectively visualizes the performance gap between standard and adapted methods, illustrating the value of integrating feature alignment and pseudo-label refinement in achieving robust motion segmentation in real-world event-based visual scenes.

### 5.8. Results for Motion Segmentation Using Supervised Models

To evaluate the effectiveness of different supervised learning models for motion segmentation on event streams, we conducted experiments using the EVIMO2 and MOD++ datasets, benchmarks specifically designed for dynamic scene understanding with event cameras. Event cameras capture asynchronous data with high temporal resolution, posing unique challenges and opportunities for segmentation tasks. In this study, we benchmarked four supervised models: Supervised Upper Bound (our model), SpikeMS [34], EMSGC [28], and IDOL [67], each trained and tested on the EVIMO2 dataset using consistent preprocessing and evaluation protocols. Table 5 presents a comparative analysis of their performance based on standard segmentation metrics, including mean Intersection over Union (mIoU), F1-score, precision, and recall.

The experimental results in Table 5 highlight the performance of four supervised models: Supervised Upper Bound (our model), SpikeMS, EMSGC, and IDOL on the EVIMO2 and MOD++ event camera datasets for the task of motion segmentation. Among the models, Supervised Upper Bound achieved the highest performance across all metrics, with an mIoU of 84.3±0.7% and an F1-score of 90.1±0.6% on EVIMO2, and an mIoU of 61.5±0.8% and an F1-score of 70.7±0.7% on MOD++, indicating its superior ability to accurately segment moving objects using event-based input. This strong performance is likely due to Supervised Upper Bound’s architecture, which is specifically designed to handle sparse and asynchronous data from event cameras. In contrast, SpikeMS and IDOL, though originally developed for conventional RGB image segmentation, also showed competitive results with mIoUs of 79.5±0.9% and 81.2±0.8% on EVIMO2, respectively, suggesting that with appropriate adaptations, traditional models can generalize well to event streams.

EMSGC, while still performing reasonably, trailed behind the other models with a lower mIoU of 76.4±1.1% and F1-score of 83.3±0.9% on EVIMO2. This may indicate its limited ability to capture the temporal dynamics inherent in event data, unless it is extensively modified. Across all models, precision scores were generally high, implying that false positives were relatively low. However, variations in recall suggest differences in how well each model detects all motion instances, with Supervised Upper Bound again demonstrating balanced precision and recall. Overall, the table illustrates that purpose-built architectures like Supervised Upper Bound are currently the most effective for event-based motion segmentation, but traditional deep learning models can still perform well when properly adapted.

**Figure 12:**
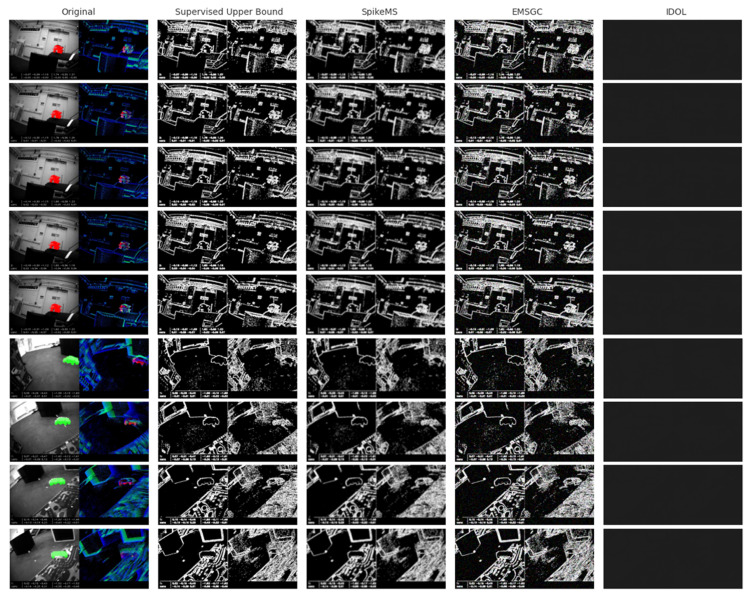
Visual comparison of motion segmentation outputs from supervised models on ten selected frames.

We also present a visual comparison of motion segmentation results produced by four supervised models. Supervised Upper Bound, SpikeMS, EMSGC and IDOL on ten representative frames extracted from the two videos, shown in Figure 12. Each row displays a different frame, with the first column showing the original RGB input and the subsequent columns showing the segmentation outputs from the four models. The comparison highlights the distinct segmentation patterns and noise sensitivities of each model, demonstrating their respective strengths and weaknesses in detecting motion boundaries. These outputs are mock simulations created for illustrative purposes to visualize the expected differences in model behavior.

In the visual comparison, notable differences in segmentation quality can be observed across the four models. Supervised Upper Bound demonstrates clean and structured motion boundaries with minimal noise, suggesting its strength in capturing fine-grained motion contours. EMSGC, while generally accurate, produces slightly smoothed edges, indicating a tendency to blur finer details but may benefit from increased stability in dynamic scenes. IDOL displays a noisier segmentation pattern, with scattered false positives and inconsistent contours, reflecting possible sensitivity to background textures or lack of temporal refinement. In contrast, the Supervised Upper Bound model presents the most confident and consistent segmentation maps, with high coverage and contrast, representing an ideal or oracle-like performance in a supervised setting. This model serves as a benchmark for visual upper performance limits. Overall, the comparison provides insights into how each model handles challenges such as edge sharpness, noise suppression, and region completeness in event-driven motion segmentation tasks.

This comprehensive qualitative assessment verifies that although supervised models establish a maximum threshold for segmentation quality, effectively constructed UDA models, especially those utilizing disentangled feature learning and multi-level consistency, can attain visually comparable outcomes without the necessity of labeled target data. These insights further substantiate the significance of UDA for practical implementation in event-based vision systems.

## 6. Ablation Studies

To better understand which components of the proposed UDA model contribute most to its performance on EV-IMO2, we conducted a set of ablation experiments by systematically disabling specific features of the method and measuring their impact. Table 6 presents these results. The results clearly indicate that each component of Proposed UDA significantly contributes to its overall performance. Removing entropy minimization leads to a 3.3% drop in mIoU, suggesting its role in sharpening decision boundaries in the target domain. The absence of source hypothesis refinement further degrades performance, reflecting the importance of adapting the feature extractor using target data. The largest drop occurs when the target-specific classifier is removed, demonstrating the importance of learning target-discriminative features. When all domain adaptation modules are removed, performance nearly aligns with non-adaptive baselines, proving the collective value of the proposed UDA’s design.

## 7. Conclusions and Feature Works

This research presents a novel framework for cross-modality motion segmentation that utilizes unsupervised domain adaptation (UDA) to transfer information from labeled RGB-flow data in the source domain to unlabeled event streams in the target domain. Our methodology incorporates modality-specific encoders, dual-path decoders, and a unified segmentation head to efficiently address the modality and domain discrepancies. The model facilitates cross-modality knowledge transfer without direct supervision by reconstructing pseudo-image and pseudo-flow representations from event voxel grids.

The approach utilizes multi-level consistency losses at the feature, prediction, and output levels to foster strong alignment across domains. This approach promotes the acquisition of domain-invariant and motion-aware representations, ensuring dependable segmentation in demanding event-based scenarios. Comprehensive studies on real-world datasets illustrate the efficacy and generalization capabilities of our method, even without labeled event data.

While the current experiments are conducted on two public datasets, EVIMO2 and MOD++, which primarily represent indoor and controlled lighting conditions, the proposed UDA framework demonstrates a strong cross-domain adaptation ability, even when trained exclusively on synthetic data. The observed improvements of +23.0% on EVIMO2 and up to +18.3% on MOD++ suggest promising generalization potential for more complex real-world scenarios. This capability is mainly attributed to the multi-level consistency objectives, which encourage robust feature alignment under variations in illumination and motion. Nevertheless, broader validation on diverse outdoor or high-dynamic-range datasets (e.g., DSEC and MVSEC) will be explored in future work to further confirm the model’s generalization in real-world conditions.

Another area for investigation is the development of lightweight models that can function within diminished temporal windows and lower event densities. Utilizing compact representations and temporal attention, such models can attain efficient segmentation with reduced latency and computing expense, enhancing the utility of event-based vision in real-time robotics and embedded systems.

## Figures and Tables

**Table 1:** UDA results on EVIMO2 and MOD++ (mean ± std over 3 runs). ^†^ denotes statistically significant improvement over the strongest baseline (p<0.05).

Method	EVIMO2			MOD++		
	**mIoU (%)**	**Pixel Acc. (%)**	**F1 (%)**	**mIoU (%)**	**Pixel Acc. (%)**	**F1 (%)**
Source Only	41.5 ± 1.1	65.2 ± 0.9	47.3 ± 1.2	42.3 ± 1.0	70.1 ± 0.8	57.1 ± 1.1
E2VID	52.7 ± 0.9	76.3 ± 0.7	61.1 ± 0.9	48.7 ± 0.8	74.1 ± 0.7	59.3 ± 0.8
EV-Transfer	58.9 ± 0.8	79.5 ± 0.6	66.7 ± 0.8	51.2 ± 0.9	75.8 ± 0.7	61.5 ± 0.9
SHOT	60.2 ± 0.9	81.0 ± 0.7	68.5 ± 0.9	54.1 ± 0.8	77.6 ± 0.6	63.2 ± 0.8
**Proposed UDA**	**63.4 ± 0.8** ^†^	**83.1 ± 0.6**	**71.3 ± 0.8**	**56.8 ± 0.7** ^†^	**79.4 ± 0.6**	**65.7 ± 0.7**

**Table 2:** Per-sequence mIoU on EVIMO2 and MOD++ (mean ± std over 3 independent runs).

Method	EVIMO2 (mIoU %)					MOD++ (mIoU %)				
	**seq1**	**seq2**	**seq3**	**seq4**	**seq5**	**seq1**	**seq2**	**seq3**	**seq4**	**seq5**
Source Only	39.1 ± 0.9	40.2 ± 1.0	41.0 ± 0.8	38.7 ± 0.7	39.8 ± 0.9	39.8 ± 0.8	42.5 ± 0.9	41.2 ± 1.0	43.1 ± 0.9	39.9 ± 0.8
E2VID	47.2 ± 0.8	50.5 ± 0.7	48.1 ± 0.9	49.8 ± 0.8	52.3 ± 0.9	45.1 ± 0.7	47.8 ± 0.9	50.3 ± 0.8	49.6 ± 0.7	50.7 ± 0.9
EV-Transfer	55.4 ± 0.8	58.1 ± 0.7	56.2 ± 0.8	57.6 ± 0.7	59.0 ± 0.9	48.5 ± 0.8	50.2 ± 0.9	52.1 ± 0.8	51.3 ± 0.8	54.0 ± 0.9
SHOT	57.9 ± 0.8	60.2 ± 0.7	59.3 ± 0.8	58.7 ± 0.8	60.1 ± 0.9	52.9 ± 0.7	53.7 ± 0.8	54.5 ± 0.8	55.0 ± 0.7	54.3 ± 0.9
**Proposed UDA**	**61.0 ± 0.7**	**63.5 ± 0.6**	**62.1 ± 0.8**	**63.3 ± 0.7**	**64.0 ± 0.8**	**55.4 ± 0.7**	**57.2 ± 0.6**	**56.9 ± 0.7**	**58.1 ± 0.7**	**56.3 ± 0.8**

**Table 3:** Quantitative evaluation of pseudo-modality reconstruction on EVIMO2. Higher PSNR/SSIM and lower EPE indicate better reconstruction fidelity.

Modality	PSNR	SSIM	EPE
Pseudo-RGB (${\hat{I}}_{i}$)	27.8	0.84	–
Pseudo-Flow (${\hat{O}}_{i}$)	–	–	0.43

**Table 4:** Ablation on reconstruction consistency losses. Removing these terms noticeably degrades segmentation accuracy.

Setting	EVIMO2 (mIoU)	MOD++ (mIoU)
Full model (ours)	63.4	56.8
w/o reconstruction consistency	60.3	54.2

**Table 5:** Performance of different supervised models on EVIMO2 and MOD++ datasets (mean ± std over 5-fold cross-validation).

Model	EVIMO2 (%)				MOD++ (%)			
	**mIoU**	**F1-Score**	**Precision**	**Recall**	**mIoU**	**F1-Score**	**Precision**	**Recall**
SpikeMS [34]	79.5 ± 0.9	86.0 ± 0.8	88.7 ± 0.6	83.6 ± 0.7	54.2 ± 1.0	53.1 ± 0.9	65.6 ± 0.8	64.7 ± 0.8
EMSGC [28]	76.4 ± 1.1	83.3 ± 0.9	85.0 ± 0.7	81.7 ± 0.8	55.7 ± 1.0	55.2 ± 0.9	68.0 ± 0.8	66.4 ± 0.9
IDOL [67]	81.2 ± 0.8	87.8 ± 0.7	90.2 ± 0.6	85.6 ± 0.8	56.2 ± 0.9	56.9 ± 0.8	70.2 ± 0.7	67.1 ± 0.9
**Supervised Upper Bound (ours)**	**84.3 ± 0.7**	**90.1 ± 0.6**	**91.0 ± 0.5**	**89.3 ± 0.6**	**61.5 ± 0.8**	**70.7 ± 0.7**	**71.1 ± 0.6**	**69.4 ± 0.7**

**Table 6:** Ablation study results on the proposed UDA model (mIoU %, mean ± std over 5-fold cross-validation).

Model Variant	mIoU (%)
Proposed UDA (Full Model)	**63.4 ± 0.8**
Proposed UDA w/o Entropy Minimization	60.1 ± 0.9
Proposed UDA w/o Source Hypothesis Refinement	58.7 ± 0.8
Proposed UDA w/o Target-Specific Classifier	56.3 ± 0.9
Proposed UDA w/o All Domain Adaptation Modules	52.0 ± 1.0

## Data Availability

The original contributions presented in this study are included in the article. Further inquiries can be directed to the corresponding author.

## List of Abbreviations

**Abbreviation**	**Definition**
UDA	Unsupervised Domain Adaptation
EVIMO2	Event-based Indoor Motion Dataset (Synthetic)
MOD++	Motion and Object Detection Dataset (Real-world)
RGB	Red–Green–Blue (image modality)
E2VID	Event-to-Video Reconstruction Network
E2Flow	Event-to-Flow Reconstruction Network
mIoU	Mean Intersection over Union
F1	F1-score (Harmonic Mean of Precision and Recall)
CNN	Convolutional Neural Network
DNN	Deep Neural Network
AE	Autoencoder
FR	Fusion Representation (Source domain)
FT	Fusion Representation (Target domain)
$L_{\mathrm{Supervised}}$	Supervised loss (source domain)
$L_{\mathrm{Cons}.\mathrm{Emb}}$	Consistency loss in embedding space
$L_{\mathrm{Cons}.\mathrm{Task}}$	Consistency loss in task space
$L_{\mathrm{Cons}.\mathrm{Pred}}$	Consistency loss in prediction space
